# Physical therapy enhances physical and functional abilities in a female patient with gliomatosis cerebri

**DOI:** 10.23938/ASSN.1087

**Published:** 2024-08-22

**Authors:** Elena Lozano-Cavero, Alfredo Lerín-Calvo, Patricia Martín-Casas, Adrián Arranz-Escudero

**Affiliations:** 1 Policlínica Glavic Neurorehabilitación Madrid España; 2 Neuron Centro de rehabilitación neurológica Madrid España; 3 Universidad Autónoma de Madrid Centro Superior de Estudios Universitarios La Salle Departamento de Fisioterapia Madrid España; 4 Universidad Complutense de Madrid Facultad de Enfermería, Fisioterapia y Podología Departamento de Radiología, Rehabilitación y Fisioterapia Madrid España; 5 Instituto de Investigación Sanitaria del Hospital Clínico San Carlos (IdISCC) Madrid España

**Keywords:** Exercise Therapy, Physical Therapy Modalities, Neoplasms Neuroepithelial, Neurological Rehabilitation, Terapia por Ejercicio, Modalidades de Fisioterapia, Neoplasias Neuroepiteliales, Rehabilitación Neurológica

## Abstract

Gliomatosis cerebri is a low incidence diffuse glial tumor that affects three or more brain lobes. The 5-year survival rate is <20%. While chemotherapy may extend life expectancy, no interventions have been documented to improve patient´s quality of life, suggesting that physical therapy might offer potential benefits in this regard.

We present the case of a female patient with gliomatosis cerebri of nine years of evolution showing dependence on technical supports and supervision during walking, reduced speed and balance, tremor and dystonia in lower limbs. The patient underwent physiotherapy treatment: trunk control exercises, limb strength, and gait ability. Improvement in functionality was observed. Further studies with a larger sample size are needed to confirm these results.

## RESUMEN

La gliomatosis cerebri es un tumor glial difuso que afecta a tres o más lóbulos cerebrales, con una incidencia baja y un pronóstico de supervivencia a 5 años del 18,8%. A pesar de que la quimioterapia parece mejorar la esperanza de vida, no se han descrito intervenciones que mejoren la calidad de vida de los pacientes, por lo que la fisioterapia podría contribuir a ello.

Se presenta el caso de una paciente con gliomatosis cerebri de nueve años de evolución con dependencia de apoyos técnicos y supervisión durante la marcha, velocidad y equilibrio reducidos, temblor y distonía en miembros inferiores. Se llevó a cabo un programa de fisioterapia basado en ejercicios de fuerza de miembros inferiores y tronco, fuerza y capacidad de marcha que resultó eficaz para mejorar la funcionalidad de esta paciente. No obstante, se necesitan ensayos clínicos con participantes similares para comprobarlo.

## INTRODUCTION

Gliomatosis cerebri (GC) is a diffuse glial tumor that invades the cerebral white matter. It exhibits an infiltrative growth pattern and typically affects three or more cerebral lobes^1^. It is considered a rare primary tumor, representing less than 1% of all astrocytomas. Due to its aggressive behavior, characterized by rapid growth and resistance to treatment, the World Health Organization classifies it as of Grade III malignancy^1^. Its overall incidence is 40 new cases per year, with a five-year survival rate of 18.8%^2^.

Compared to surgery or radiotherapy alone, chemotherapy has proven to be the most effective option for extending the life expectancy of GC patients, bringing their survival closer to that of individuals with Grade II or III glioma^3^. Physiotherapy may have positive outcomes for people with glioma, as moderate-intensity exercise significantly inhibits cancer cell proliferation and strengthens the immune system^4^. To date, there are no publications on physiotherapy interventions for patients with CG. The purpose of this clinical note was to contribute data on the treatment of a patient with this condition and explore the potential effects of physiotherapy on motor control, balance, strength, and gait performance.

## CASE REPORT

We present the case of a 43-year-old Caucasian woman who attended a neurological rehabilitation center in Madrid in October 2022 due to progressively worsening neurological symptoms. She was initially diagnosed in 2013 with infiltrative GC affecting both pyramidal tracts and the lemniscus. The patient explained that her deteriorating condition, coupled with confinement due to COVID-19, contributed to the significant delay between her diagnosis and her first evaluation at the center.

Since 2014, she has experienced mild action tremor in her upper limbs and neck, which worsened with antidepressant use. By 2019, she began showing symptoms of progressive gait disorder accompanied by numbness sensation in the feet ascending to the infraumbilical area, bilateral Achilles clonus, level 2 spasticity on the modified Ashworth scale, bilateral kick sign, unstable Romberg and postural, vocal, and cephalic tremors, and sensory ataxia. No pharmacological treatment was administered prior to or during rehabilitation.

During her initial physical therapy evaluation, she struggled to stand and required a crutch for support while walking. She exhibited knee locking in extension, along with imbalance and global tremor, which was less pronounced when seated. This led to a score of three out of five on the Functional Ambulation Categories scale. Notably, she reported experiencing no noticeable fatigue or falls.

Balance, gait and endurance were assessed at admission and after three and five months of treatment using the following clinical scales:

- Timed Up and Go (TUG): assesses postural changes and gait by counting the seconds to get up from the seat, walk three meters, and sit down again; > 20 s indicates high risk of falling;
- 10 Meter-Walk Test (10MWT): evaluates walking speed based on the seconds it takes to walk 10 meters; > 15 s indicates high risk of falling;
- 6 Minute-Walk Test (6MWT): evaluates aerobic capacity and walking by recording the distance covered in 6 minutes;
- Berg Balance Scale (BBS): assesses static balance and changes in the position of the center of gravity through fourteen tasks that are scored according to a scale, from 0 (disability) to 4 (maximum capacity); the maximum score is 56 points and < 20 indicates high risk of falling;
- Functional Gait Assessment (FGA): evaluates balance and coordination during walking through 10 tasks scored on a scale from 0 (great difficulty) to 3 (complete ability). The maximum possible score is 30 points, with a score of less than 22 indicating high risk of falling.

The results indicated limited ability to walk long distances, average aerobic capacity, moderate/high risk of falls, and need for verbal supervision (Table 1).

**Table 1:** Results of balance, gait, and endurance assessments at admission and at three and five months of treatment

Assessment	Evaluation		
	Admission	3 months	5 months
	10/26/2022	01/25/2023	03/25/2023
TUG	10.32 s	10.18 s	11.05 s
10mWT	0.63 m/s	0.54 m/s	0.64 m/s
6MWT	193.5 m	214.7 m	200.0 m
BBS	42/56	50/56	50/56
FGA	7/30	17/30	23/30

TUG: Timed Up and Go; 10MWT: 10 Meter Walk Test; 6MWT: 6 Minute Walk Test; BBS: Berg Balance Scale; FGA: Functional Gait Assessment; m: meters; s: seconds

The therapeutic goals established collaboratively between the physical therapist and the patient were: 1) complete the 6MWT without a crutch; 2) wear 4-centimeter high heeled boots to an event; 3) walk 1 km outdoors without breaks, using a crutch; 4) walk accompanied using the crutch to her workplace on three separate days; and 5) get up once daily to walk without a crutch to the coffee machine or bathroom at work.

The patient received one-hour physiotherapy sessions based on therapeutic exercise three times a week for five months. The treatment plan integrated proprioception exercises and both static and dynamic balance training. This included maintaining various positions and walking on unstable surfaces, avoiding obstacles, disturbances in the shoulder girdle or spine. Additionally, the Tymo robotic platform was utilized to enhance the exercises. Coordination through rhythmic exercises performed in supine, quadruped, or standing position alternating tasks and actions. Strengthening the lumbopelvic and abdominal muscles through exercises that activate the abdominal muscles while seated on an exercise ball or in a supine position; these exercises involved performing movements that cross the midline of the body. Strengthening of the lower limbs through strength work with progressive resistance exercises of the quadriceps, gluteus medius, gluteus maximus, hamstrings, and tibia-lis anterior muscles. Aerobic exercise using a treadmill and cycloergometer at moderate intensity progressively increasing over time to 60% of the patient´s maximum heart rate. The intensity and difficulty of the exercises were adjusted progressively based on the patient´s improvements.

The patient’s adherence to treatment was adequate: she attended all 50 scheduled sessions. She did not perform any additional exercises at home.

The achievement of the patient’s personal goals was assessed with Goal Attainment Scaling with outcomes rated as follows: exceeded as described (0), better than expected (+1), better and earlier than expected (+2) or, conversely, her performance worsened somewhat (-1) or worsened considerably (-2). The unfavorable results in Table 2 may be biased by external factors that should be considered such as rainy weather and an ankle sprain affecting objective 2 or the lack of a companion for objective 4.

Significant improvements were also noted in daily functional activities that were not been initially set as goals. The patient was able to take a standing shower, pick up objects from the floor, walk 3 km with breaks every kilometer, shop for half an hour, use escalators, and return to work in an office role, an achievement the patient described as highly significant.

At the end of the fifth month of treatment, the patient informed us that she would discontinue rehabilitation indefinitely for personal reasons. She expressed her intention to continue following the recommendations and exercises as much as possible. A follow-up attempt was made two months later, but no response was received, leaving her current status unknown.

**Table 2:** Goal Attainment Scaling assessment of objectives at five months

GAS goals	Rating
1) Walking indoors for 6 min (6MWT) using a crutch	1
2) Wearing boots to social events, including lunch, dinner and gatherings with friends	- 1
3) Walking 1 km outdoors using the crutch without rest breaks	0
4) Walking with a companion from home to her workplace on three different days	- 1
5) Standing and walking at her workplace without a crutch three times per week	- 1

## DISCUSSION

After five months of physiotherapy focused on muscle strength and endurance exercises, similar to those proven highly effective in ataxia and multiple sclerosis^5^^,^^6^, the changes observed in the 10MWT and 6MWT are barely detectable. In patients with multiple sclerosis, the minimum detectable changes are 0.26 m/s^7^ and 76.2 m^8^, respectively. However, the patient achieved a personal milestone by completing the 6MWT without a crutch after the second month of intervention.

In contrast to the outcomes described above, the BBS score is always consistently close to the maximum from the first evaluation, surpassing the MDC of three points for patients with ataxia^7^ and 7 points for patients with multiple sclerosis^8^. Additionally, the FGA shows significant improvement by the third evaluation, exceeding the MDC of 6 points for patients with ataxia, multiple sclerosis, and balance disorders^9^. This finding may be attributed to the correlation between these features in patients with cerebellar disease and ataxia gait^10^. Effective trunk muscle movement and axial stabilization are crucial for improved gait performance in neurological patients. Ataxia and multiple sclerosis were chosen for comparison due to their similarities in neurological symptoms and the carcinogenic etiology of the glioma.

Regarding personal goals, the patient found that the unexpected benefits gained in daily living activities are more meaningful to the patient than the goals initially set. Being able to go shopping after three years or return to work can add significant meaning to rehabilitation progress. Such achievements can enhance the patient´s sense of fulfillment, motivation, and reward, encouraging her to maintain or even increase the effort she has invested in her recovery, regardless of clinical evaluations.

In conclusion, a physical therapy program can enhance functional abilities such as balance, gait, and activities of daily living, thereby improving the quality of life for patients with gliomatosis cerebri. While randomized clinical trials are needed to confirm these findings, this patient´s case is particularly relevant given the limited evidence available, owing to the low incidence of this tumor.
